# Neuregulin-1 attenuates experimental cerebral malaria (ECM) pathogenesis by regulating ErbB4/AKT/STAT3 signaling

**DOI:** 10.1186/s12974-018-1147-z

**Published:** 2018-04-10

**Authors:** Mingli Liu, Wesley Solomon, Juan Carlos Cespedes, Nana O. Wilson, Byron Ford, Jonathan K. Stiles

**Affiliations:** 1 Department of Microbiology, Biochemistry and Immunology, Morehouse School of Medicine, 720 Westview Drive SW, Atlanta, GA 30310 USA; 2 Fogarty Global Health Fellow (UJMT), Morehouse School of Medicine, 720 Westview Drive SW, Atlanta, GA 30310 USA; 3 Division of Biomedical Sciences, University of California-Riverside School of Medicine, 900 University Ave, Riverside, CA 92521 USA

**Keywords:** Cerebral malaria (CM), Neuregulin-1 (NRG-1), ErbB4, STAT3, AKT, *P. berghei* ANKA (PbA)

## Abstract

**Background:**

Human cerebral malaria (HCM) is a severe form of malaria characterized by sequestration of infected erythrocytes (IRBCs) in brain microvessels, increased levels of circulating free heme and pro-inflammatory cytokines and chemokines, brain swelling, vascular dysfunction, coma, and increased mortality. Neuregulin-1β (NRG-1) encoded by the gene *NRG1*, is a member of a family of polypeptide growth factors required for normal development of the nervous system and the heart. Utilizing an experimental cerebral malaria (ECM) model (*Plasmodium berghei* ANKA in C57BL/6), we reported that NRG-1 played a cytoprotective role in ECM and that circulating levels were inversely correlated with ECM severity. Intravenous infusion of NRG-1 reduced ECM mortality in mice by promoting a robust anti-inflammatory response coupled with reduction in accumulation of IRBCs in microvessels and reduced tissue damage.

**Methods:**

In the current study, we examined how NRG-1 treatment attenuates pathogenesis and mortality associated with ECM. We examined whether NRG-1 protects against CXCL10- and heme-induced apoptosis using human brain microvascular endothelial (hCMEC/D3) cells and M059K neuroglial cells. hCMEC/D3 cells grown in a monolayer and a co-culture system with 30 μM heme and NRG-1 (100 ng/ml) were used to examine the role of NRG-1 on blood brain barrier (BBB) integrity. Using the in vivo ECM model, we examined whether the reduction of mortality was associated with the activation of ErbB4 and AKT and inactivation of STAT3 signaling pathways. For data analysis, unpaired *t* test or one-way ANOVA with Dunnett’s or Bonferroni’s post test was applied.

**Results:**

We determined that NRG-1 protects against cell death/apoptosis of human brain microvascular endothelial cells and neroglial cells, the two major components of BBB. NRG-1 treatment improved heme-induced disruption of the in vitro BBB model consisting of hCMEC/D3 and human M059K cells. In the ECM murine model, NRG-1 treatment stimulated ErbB4 phosphorylation (pErbB4) followed by activation of AKT and inactivation of STAT3, which attenuated ECM mortality.

**Conclusions:**

Our results indicate a potential pathway by which NRG-1 treatment maintains BBB integrity in vitro, attenuates ECM-induced tissue injury, and reduces mortality. Furthermore, we postulate that augmenting NRG-1 during ECM therapy may be an effective adjunctive therapy to reduce CNS tissue injury and potentially increase the effectiveness of current anti-malaria therapy against human cerebral malaria (HCM).

## Background

Human cerebral malaria (HCM) is a severe form of malaria characterized by sequestration of infected erythrocytes (IRBCs) in brain microvessels, increased levels of circulating free heme and pro-inflammatory cytokines and chemokines, brain swelling, vascular dysfunction, coma, and increased mortality. The resulting leakiness of the blood brain barrier (BBB) caused by the decreased cerebralvascular integrity allows increased trafficking of toxic compounds into the brain parenchyma leading to exacerbation of neurological deficits [1, 2]. The BBB is a highly selective semipermeable membrane barrier consisting of cerebral vascular endothelial cells and astrocytes surrounding them. It separates the circulating blood from the brain and extracellular fluid [3] and protects neural tissues against various unfavorable compositions and toxins in the blood. Dysfunctional microvascular endothelial cells or astrocytes compromise the integrity of the BBB, a hallmark of HCM pathogenesis [4, 5]. We have reported that elevation of circulating CXCL10 and free heme induce apoptosis of human brain microvascular endothelial cells (hCMEC/D3) and astroglia/neuroglia (M059K) [6, 7], indicating the important roles played by circulating CXCL10 and free heme in mediating experimental cerebral malaria (ECM) and HCM pathogenesis, BBB integrity, and mortality [8, 9].

The neuregulin family of ligands consist of four members, neuregulin 1β (NRG-1), NRG-2, NRG-3, and NRG-4. While little is known about the biological functions of NRG-2, NRG-3, and NRG-4 [10], NRG-1 has been widely studied in stroke [11, 12], cardiovascular diseases [13, 14], and tumors [15, 16]. NRG-1, a secreted trophic factor, is encoded by the *neuregulin/NRG-1* gene located on the short arm of chromosome 8 [17, 18]. Alternative splicing produces at least 15 different NRG-1 isoforms, which are grouped as types I, II, and III [19, 20]. All four genes in the NRG-1 family (NRG1–4) share a common epidermal growth factor (EGF)-like domain. Type I NRGα and NRGβ isoforms are the predominant isoforms expressed in early embryogenesis, whereas types II and III NRG are not detectable until at mid-gestation stage [19]. Type III, which is also called sensory and motor neuron-derived factor (SMDF), is the most dominant type of NRG-1 in the human adult brain, accounting for about 73% of total NRG-1 [21, 22]. The ErbB receptors are a family composed of receptors of ErbB1, ErbB2, ErbB3, and ErbB4. Any isoform of NRG1 is capable of directly binding and activating ErbB3 and ErbB4 receptors although the biological significance is incompletely understood [19]. The ErbB3 receptor lacks an active kinase domain and is unable to form functional ErbB3 homodimers [23]. ErbB4 undergoes tertiary structural changes in the juxtaembrane region when it binds to its ligand NRG-1 and forms functional homodimers or heterodimers with ErbB1, ErbB2, and ErbB3. Phosphorylation of the ErbB4 receptor results in the recruitment of adaptor/effecter molecules [24] and initiates/activates numerous downstream signaling pathways crucial to neuronal development, neuronal migration, axonal navigation, and synaptic function [25]. We recently reported that intravenous infusion of NRG-1 significantly reduced mortality associated with ECM by promoting a robust anti-inflammatory response and reducing accumulation of intra vascular IRBC as well as tissue damage [26]. In the current study, we explored a potential molecular mechanism by which NRG-1 regulates downstream pathways to improve/restore the integrity of the BBB and attenuate ECM pathogenesis.

## Results

### NRG-1 protects against CXCL10- and heme-induced apoptosis in astrocytes and endothelial cells

**Fig. 1:**
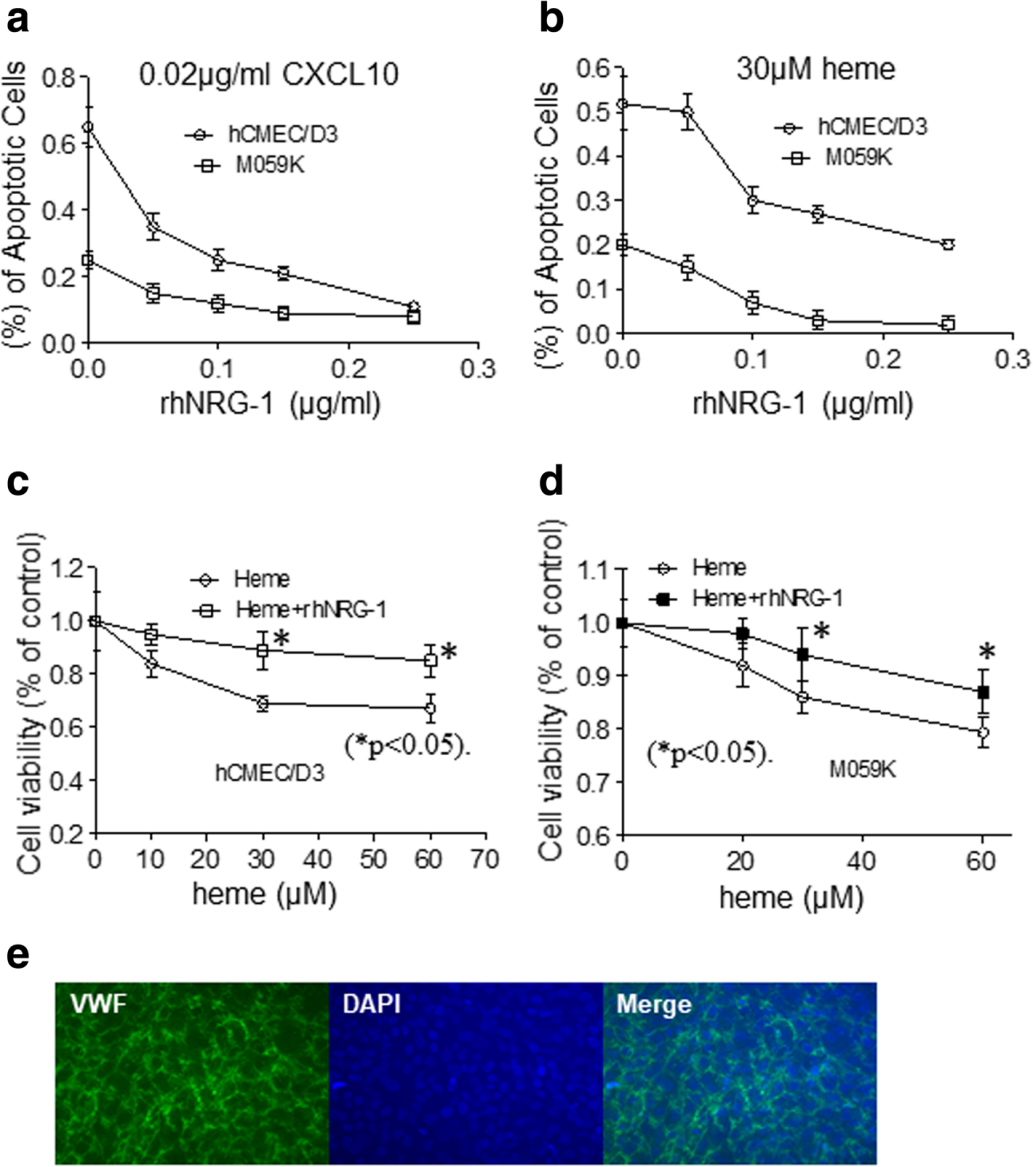
*NRG-1 protects against CXCL10*, *heme-induced astrocytes*, *and endothelial cell apoptosis*. Human brain microvascular endothelial cells (hCMEC/D3) and human neuroglial cells/astrocytes (M059 K) were treated with increasing doses of NRG-1 followed by CXCL10 or heme treatment, respectively, for 24 h. Apoptosis and cell proliferation were measured by TUNEL or MTT assay accordingly. **a** CXCL10 induces apoptosis in hCMEC/D3 and M059K cells in a dose-dependent manner. Addition of NRG-1 ranging from 50 to 250 ng/ml attenuated apoptosis in hCMEC/D3 and M059K cells. **b** 30 μM of heme-induced apoptosis in hCMEC/D3 and M059K cells around 52 and 20%, respectively. NRG-1 protected against heme-induced apoptosis of hCMEC/D3 and M059K cells in a dose-dependent manner. **c**. Treatment of hCMEC/D3 cells with heme at the concentration from 10 to 60 μM for 24 h induced 20–30% apoptosis in a dose-dependent manner, and cell apoptosis was attenuated when cells were pre-incubated with 100 ng/ml of NRG-1 (*p < 0.05). **d** Treatment of M059K cells with heme at concentration from 10 to 60 μM for 24 h induced 8.0–20.6% cell death in a dose-dependent manner, and cell apoptosis was attenuated when cells were pre-incubated with 100 ng/ml of NRG-1 (*p < 0.05). **e** The hCMEC/D3 cells expressed vWF, an endothelial cell marker

We previously reported that mortality associated with HCM correlates with increased serum CXCL10 levels and that treatment of both endothelial hCMEC/D3 and neuroglial M059K cells with CXCL10 at a physiologically relevant concentration of 0.02 μg/ml in vitro induced apoptosis [6]. To test the protective effects of NRG-1, we treated hCMEC/D3 and M059K cells, respectively, with CXCL10 and NRG-1 ranging from 50 to 250 ng/ml for 24 h and assayed for apoptosis by TUNEL. NRG-1 attenuated apoptosis of hCMEC/D3 and M059K cells induced by CXCL10 at concentrations as low as 50 ng/ml and up to a maximum effect at 250 ng/ml (Fig. 1a). As a follow-up to our first report on the induction of apoptosis by heme in human brain microvascular endothelial cells [7], we next tested whether NRG-1 had anti-apoptotic effect against hCMEC/D3 and M059K cells treated with heme. When M059K cells were treated with heme at the concentrations used for hCMEC/D3 endothelial cells [7], M059K cell apoptosis occurred at around 20%. Although apoptosis was modest compared with that in endothelial hCMEC/D3 cells (52%) induced by heme [7], protection of apoptosis by NRG-1 was significant (Fig. 1b). Furthermore, NRG-1 attenuated apoptosis of both hCMEC/D3 and M059K cells either induced by CXCL10 or heme in a dose-dependent manner. In agreement with Lok et al. [27], we used 100 ng/ml of NRG-1 in the following experiments because at this concentration more than 50% of apoptotic cells were rescued (Fig. 1a, b). We next conducted a dose response assay with heme ranging from 10 to 60 μM to assess protection of NRG-1 against heme-induced apoptosis. Both hCMEC/D3 and M059K cells were treated with 10 to 60 μM of heme along with 100 ng/ml NRG-1 for 24 h followed by measurement of cell viability using MTT assay. NRG-1 attenuated 20–30% of heme-induced hCMEC/D3 cell death at each heme concentration (Fig. 1c). Similar observations were made when neuroglial M059K cells were treated with different doses of heme, reducing 8 to 20.6% cell death in the presence of 100 ng/ml of NRG-1 for 24 h (Fig. 1d). Figure 1e indicated that hCMEC/D3 used in the experiments express the characteristic endothelial cell marker von Willebrand factor VIII (VWF).

### NRG-1 attenuates heme-induced tight junction disruption in an in vitro BBB model

**Fig. 2:**
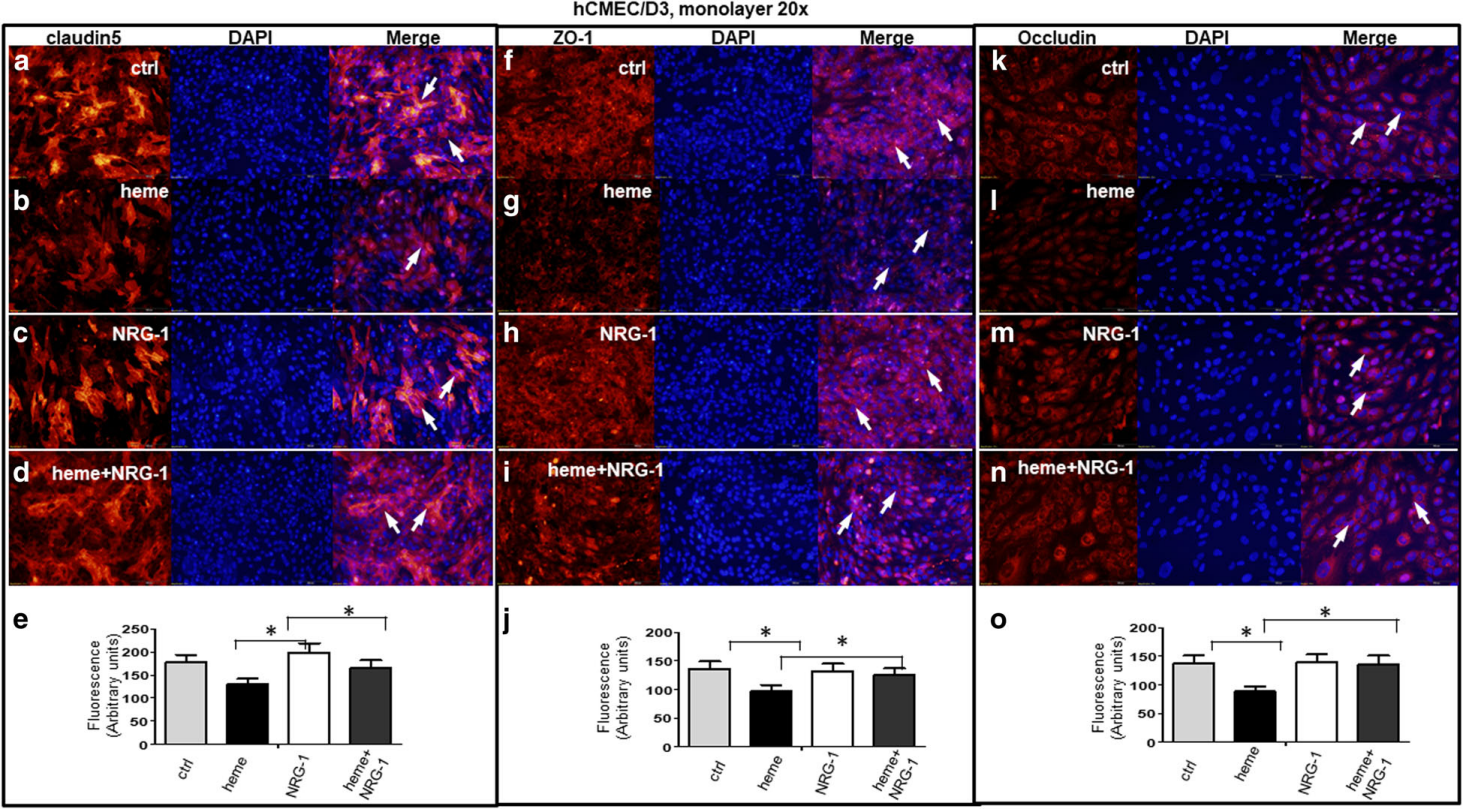
*NRG-1 attenuates heme-induced damage of human brain microvascular endothelial cells (hCMEC/D3).* **a**–**d** A monolayer of hCMEC/D3 cells were grown confluently and treated with 30 μM heme and NRG-1100 ng/ml followed by staining with anti-claudin5 antibody against tight junction protein claudin5. Claudin5 was expressed on cell membranes (arrows) which disappeared after the cells were treated with 30 μM heme for 24 h. NRG-1 treatment inhibited the disruption of heme-induced Claudin5 membrane expression. **e** Claudin5 antibody immunoreactive signals in hCMEC/D3 cells were quantitatively analyzed under each treatment as indicated. The fluorescence intensity (red) of cells was measured in 500 cells per treatment using an ImageJ software program (Version 1.51j8, National Institutes of Health (NIH)). The average relative intensity of cells associated with claudin5 antibody was 177.44 ± 16.45 in controls. Heme treatment reduced the average intensity to 129.33 ± 13.16 (*p* < 0.05). In contrast, NRG-1 treatment increased the average fluorescence intensity back to 165.23 ± 17.11 (*p* < 0.05). The asterisks indicate significant difference with a *p* value of 0.05 as determined with Student’s paired *t* test. **f**–**i** hCMEC/D3 cells were grown in monolayer treated with 30 μM heme and 100 ng/ml NRG-1 and stained with anti ZO-1 antibody against tight junction protein ZO-1. ZO-1 is expressed on cell membranes (arrows) and disappeared after cells were treated with 30 μM heme for 24 h. NRG-1 treatment inhibited disruption of heme-induced ZO-1 membrane expression. **j** Quantitation of ZO-1 staining from samples of hCMEC/D3 cells treated with heme, NRG-1, combination of heme and NRG-1, and corresponding control. Total immunofluorescence intensity with ZO-1 antibody (red) was determined with ImageJ software program, and the average values for the indicated number of cells (*n* = 500) examined were as shown. Briefly, the average relative intensity of ZO-1 of heme-treated cells decreased to 96.99 ± 10.35 as compared to controls 136.16 ± 13.00 (*p* < 0.05). The average immunofluorescence intensity with ZO-1 antibody increased to 125.45 ± 11.78 when NRG-1 was added to heme-treated cells (*p* < 0.05). The asterisks indicate significant difference with a *p* value of 0.05 as determined with Student’s paired *t* test. **k**–**n** hCMEC/D3 cells were grown in monolayer treated with 30 μM of heme and NRG-1100 ng/ml and thereafter stained with anti-occludin antibody. Occludin is expressed on cell membranes (arrows) and disappeared after cells were treated with 30 μM heme for 24 h. NRG-1 treatment prevented the disruption of heme-induced occludin expression. **o** The average relative intensity of occludin in heme-treated cells decreased to 88.04 ± 9.24 compared to controls 137.34 ± 14.23 (*p* < 0.05), while the average immunofluorescence intensity increased to 136.00 ± 15.00 when NRG-1 was added to heme-treated cells (*p* < 0.05). The asterisks indicate significant difference with a *p* value of 0.05 as determined with Student’s paired *t* test

The BBB functions as a barrier to restrict movement of potentially toxic substances from the circulation to the central nervous system [28]. It has been demonstrated that a polymerized cell-free Hb (HbG) (extracellular Hb) triggers BBB disruption and oxidative stress in a guinea pig exchange transfusion model [29]. However, it is unclear whether hemoglobin-derived heme, which induces cytotoxic oxidative stress, alters the permeability/integrity of BBB and/or disrupts intercellular tight junctions (TJs) of endothelia in addition to promoting endothelial cell and astrocyte apoptosis. We treated hCMEC/D3 cells grown in a monolayer with 30 μM heme and NRG-1 (100 ng/ml) followed by staining with antibodies against tight junction proteins claudin5, ZO-1, and occludin. Claudin5 was located on cell membranes (arrows) while the membrane-bound staining disappeared after the cells were treated with 30 μM heme for 24 h (Fig. 2a, b). NRG-1 treatment prevented reduction of membrane-bound claudin5 by heme (Fig. 2c, d). The fluorescence intensity of claudin5 signals in hCMEC/D3 cells treated with heme, NRG-1, heme/NRG-1, and corresponding control were quantitatively analyzed in 500 cells per treatment using an ImageJ software program (Version 1.51j8, National Institutes of Health (NIH)). The average relative intensity of cells was 177.44 ± 16.45 in controls. The average immunofluorescence intensity associated with claudin5 antibody of cells treated with heme was 129.33 ± 13.16 (*p* < 0.05). In contrast, the average immunofluorescence intensity with claudin5 antibody increased to 165.23 ± 17.11 when NRG-1 was added to heme-treated cells (*p* < 0.05, Fig. 2e). Similar observations were obtained with ZO-1 (Fig. 2f–i) as those obtained with claudin5. The average relative intensity of ZO-1 of heme-treated cells decreased to 96.99 ± 10.35 as compared to controls 136.16 ± 13.00 (*p* < 0.05, Fig. 2j). The average immunofluorescence intensity with ZO-1 antibody increased to 125.45 ± 11.78 when NRG-1 was added to heme-treated cells (*p* < 0.05, Fig. 2j). The expression of occludin (Fig. 2k–n) showed the same pattern as that of claudin5 and ZO-1. The average relative intensity of occludin in heme-treated cells decreased to 88.04 ± 9.24 compared to controls 137.34 ± 14.23 (*p* < 0.05). The average immunofluorescence intensity of occludin antibody increased to 144.10 ± 15.00 when NRG-1 was added to heme-treated cells (*p* < 0.05, Fig. 2o). These results suggest that NRG-1 protects against heme-induced damage of tight junction integrity in the BBB.

**Fig. 3:**
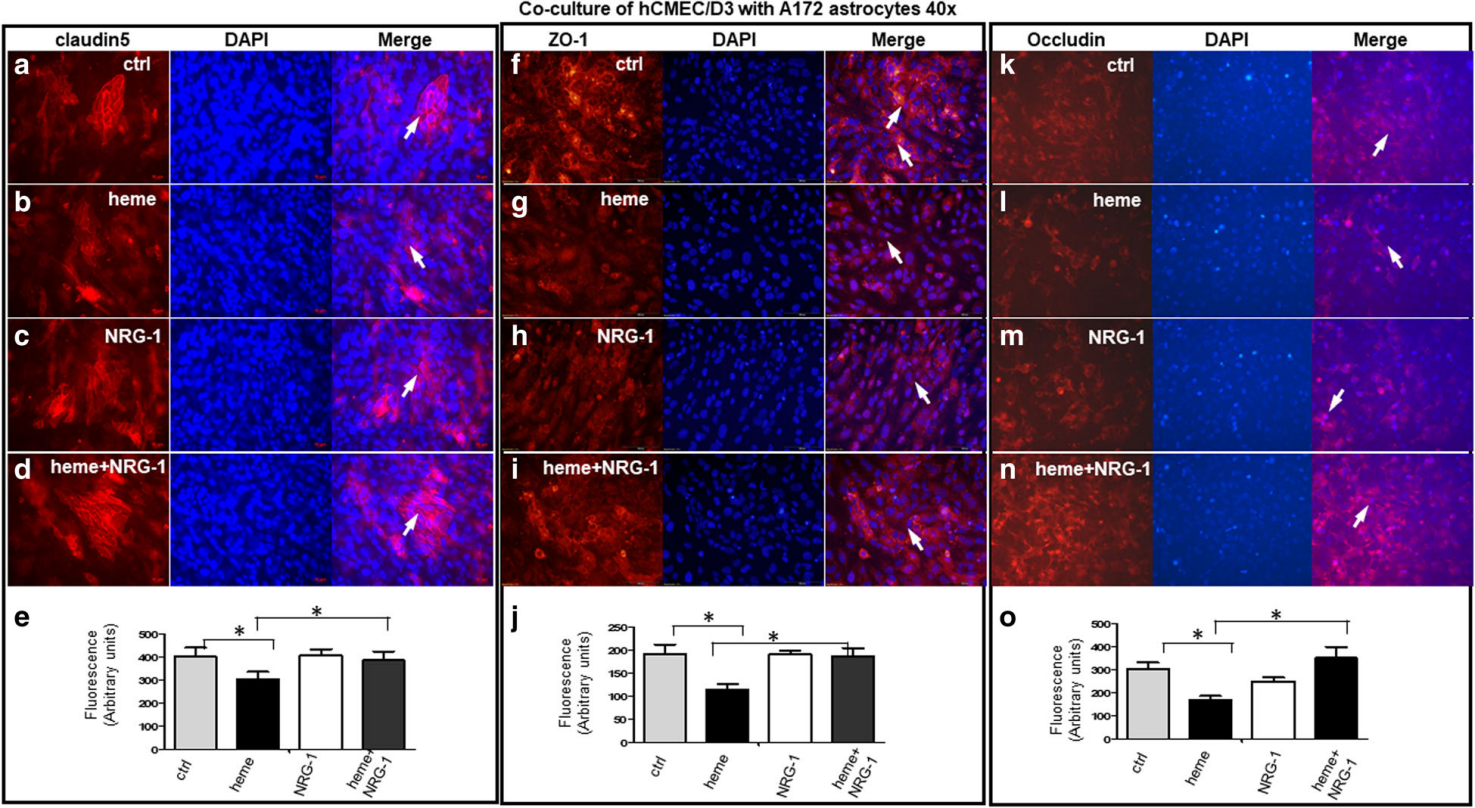
*NRG-1 attenuates heme-induced damage of BBB in an* in vitro *BBB model consisting of human brain microvascular endothelial cells (hCMEC/D3) and neuroglial cells/astrocytes of human A172 glioma cells*. **a**–**d** When hCMEC/D3 was co-cultured with human neuroglial cells/ astrocytes (human A172 glioma), Claudin 5 was expressed on cell membranes (arrows) and disappeared following treatment with 30 μM heme for 24 h. Additionally, heme reduced Claudin5 expression while NRG-1 attenuated heme-induced reduction in claudin5 expression. **e** Quantitation of claudin5 staining of hCMEC/D3 co-cultured with A172 astrocytes by different treatment as indicated. Total immunofluorescence intensity with claudin5 antibody (red) was determined with ImageJ software program, and the average value for the indicated number of cells examined (*n* = 500) is shown. Quantitative analysis of immunoreactive signals of claudin5 antibody in hCMEC/D3 cells indicated decreased staining from pronounced (401.80 ± 39.00) in control to moderate (303.21 ± 32.67) after treatment with heme (*n* = 500, *p* < 0.05), while staining for claudin5 proteins in cells treated with NRG-1 increased significantly (385.00 ± 39.97, *p* < 0.05). The asterisks indicate significant difference with a *p* value of 0.05 as determined with Student’s paired *t* test. **f**–**i** When hCMEC/D3 was co-cultured with human neuroglial cells/ astrocytes (A172 glioma), tight junction protein marker ZO-1 was expressed on cell membranes (arrows). The membranous staining was disrupted and cytoplasmic staining was more dense after cells were treated with 30 μM heme for 24 h. In addition, heme reduced ZO-1 expression while NRG-1 increased ZO-1 expression reduced by heme. **j** Quantitation of ZO-1 staining of hCMEC/D3 co-cultured with A172 astrocytes by different treatment as indicated. Total immunofluorescence intensity with ZO-1antibody (red) was determined with ImageJ software program, the average value for the indicated number of cells examined (*n* = 500) is shown. Briefly, the average immunofluorescence intensity for ZO-1decreased to moderate 114.69 ± 11.60 in heme-treated cells as compared to control (192.17 ± 20.01, *p* < 0.05), while the intensity of this staining returned to pronounced levels (187.46 ± 16.78) when the cells were treated with NRG-1 (*p* < 0.05). The asterisks indicate significant difference with a *p* value of 0.05 as determined with Student’s paired *t* test. **k**–**n** When hCMEC/D3 was co-cultured with human neuroglial cells/astrocytes from human A172 glioma, tight junction protein marker occludin was expressed on cell membranes (arrows). The membrane-bound expression disappeared after cells were treated with 30 μM heme for 24 h. In addition, heme reduced occludin expression, and NRG-1 increased occludin expression reduced by heme. **o** Quantitation of occludin staining in hCMEC/D3 cells treated with heme, NRG-1, combination of heme and NRG-1, and corresponding control. Total immunofluorescence intensity with occludin antibody (red) was determined with ImageJ software program, and the average value for the indicated number of cells examined (*n* = 500) is shown. The average relative intensity of occludin in cells treated with heme decreased to 170.11 ± 15.89 as compared to control 303.34 ± 28.98 (*n* = 500, *p* < 0.05). The average immunofluorescence intensity with occludin antibody increased to 350.00 ± 47.92 when NRG-1 was added to the cells treated with heme (*p* < 0.05). Together, these results suggest that NRG-1 protects against heme-induced damage of brain vascular endothelial cells in in vitro BBB model

To further examine the role of NRG-1 on the in vivo BBB conditions, we employed a two-dimensional (2D) in vitro BBB model to promote a phenotype that more closely mimicks in vivo physiological or pathological conditions as previously described [28, 30–33]. To enable better attachment of endothelial cells to support surfaces for immunofluorescent staining, the protocol was slightly modified. In our 2D transwell co-culture system, the monolayer of hCMEC/D3 was placed in the lower surface of the filter (brain side), with the upper chamber (blood side) containing neuroglial cell cultures from human A172 glioma (Fig. 3a–d). Treatment was initiated after 7 days of co-culture between the two cell types. The endothelial cells were allowed to acquire the appropriate BBB phenotype needed for the assay and to produce cytokines or key soluble substances [34]. This syngeneic co-culture model of endothelial and neuroglia/astrocyte cells increases tight junction resistance between the endothelial cells compared to endothelial cells grown alone (one-dimensional). Expression of tight junction protein markers were assayed by staining with antibodies against tight junction proteins such as claudin5, ZO-1, and occludin. Claudin 5 was expressed on cell membranes (arrows) in co-cultured hCMEC/D3 with human neuroglia as observed with hCMEC/D3 monolayer culture. The membrane-bound expression was disrupted and/or disappeared, while cytoplasm stained more densely after cells were treated with 30 μM heme for 24 h (Fig. 3a, b, arrows). NRG-1 attenuated the disruption of claudin5 expression induced by heme (Fig. 3c, d). Quantitative analysis of immunoreactive signals of claudin5 antibody in hCMEC/D3 cells indicate decreased staining from pronounced (401.80 ± 39.00) in control to moderate (303.21 ± 32.67) after treatment with heme (*n* = 500, *p* < 0.05), while staining for claudin5 proteins in cells treated with NRG-1 (385.00 ± 39.97, *p* < 0.05, Fig. 3e) increased significantly. Similar results were observed for ZO-1(Fig. 3f–j). The average immunofluorescence intensity for ZO-1 decreased to moderate levels (114.69 ± 11.60) in heme-treated cells when compared to control (192.17 ± 20.01, *n* = 500, *p* < 0.05), while the intensity of staining returned to pronounced levels (187.46 ± 16.78) when cells were treated with NRG-1 (*p* < 0.05, Fig. 3j). The expression pattern of occludin was similar to that of claudin5 (Fig. 3k–o). The average relative intensity of occludin in cells treated with heme decreased to 170.11 ± 15.89 as compared to that in control 303.34 ± 28.98 (*n* = 500, *p* < 0.05). The average immunofluorescence intensity with occludin antibody increased to 489.24 ± 47.92 when NRG-1 was added to the cells treated with heme (*p* < 0.05, Fig. 3o).

### Effects of NRG-1 on permeability of endothelial cells

**Fig. 4:**
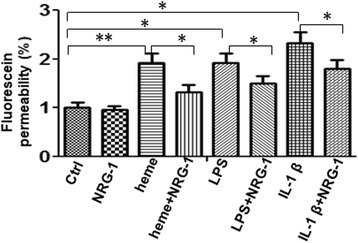
*NRG-1 reduces heme-induced endothelial cell permeability*. hCMEC/D3 cells were grown on the inner surface of gelatin-coated transwell inserts, then incubated with 30 μM heme, 10 ng/ml IL-1β, 1 μg/ml lipopolysaccharides (LPS), and 100 ng/ml NRG-1 as indicated for 24 h. Permeability was measured by adding 10 μM sodium fluorescein to the upper chamber. After incubation for 0.5 h, the sample from the lower compartment was collected and 100 μl of sample was measured for fluorescence at excitation 485 nm and emission 535 nm. Incubation with heme, LPS, and IL-1β resulted in increased permeability of the endothelial monolayer by 1.93-fold, 1.92-fold, and 2.33-fold, respectively. The presence of NRG-1 during the incubation period reduced endothelial permeability down to 1.32-fold, 1.50-fold, and 1.8-fold, respectively

BBB function can be examined by assessing permeability of confluent endothelial cells to sodium fluorescein (SF; MW = 376 kDa) [28]. Heme-induced endothelial cell permeability was assessed by growing hCMEC/D3 cells to confluence on the inner surface of gelatin-coated transwell inserts followed by incubation of cells with 30 μM heme, 10 ng/ml IL-1β, 1 μg/ml lipopolysaccharide (LPS), and 100 ng/ml NRG-1 for 24 h as indicated, respectively, while LPS and IL-1β served as positive controls. Permeability was measured by adding 10 μM sodium fluorescein to the upper chamber as previously described [35] (Fig. 4). Incubation with heme, LPS, and IL-1β resulted in increased permeability of the endothelial monolayer by 1.93-fold, 1.92-fold, and 2.33-fold, respectively. Addition of NRG-1 during incubation of the cells reduced endothelial permeability by 1.32-fold, 1.50-fold, and 1.8-fold, respectively (Fig. 4).

### Regulation of brain NRG-1 levels following ECM

**Fig. 5:**
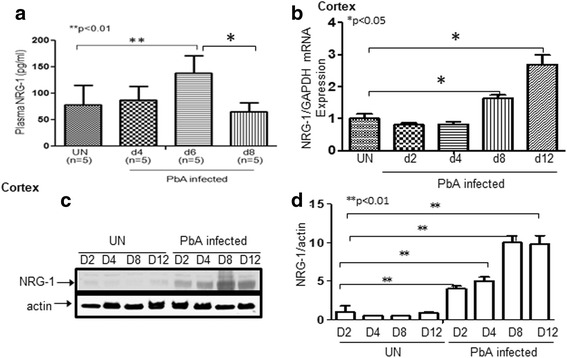
*Circulating NRG-1 levels decrease while expression levels of NRG-1 in brain tissue increase in ECM mice.* **a** Peripheral circulating blood in mice (*n* = 5/group) was assessed for total NRG-1 protein expression in serum by ELISA. Peripheral blood level of NRG-1 is significantly increased 6 days post infection in mice, but declined to a level below that of uninfected mice 8 days post infection (stage when mice begin to develop fatal brain damage). **b** Infection of C57BL/6 WT mice with PbA upregulated NRG-1 mRNA in brain tissue. **c** NRG-1 protein levels tested by Western blot analysis in the cortex were significantly increased in PbA-infected WT of C57 BL/6 mice. **d** A densitometeric analysis of bands on Western blot was performed with the ImageQuant program. The data are presented as fold stimulation relative to appropriate controls and are the mean ± SE of duplicated samples performed in three independent experiments

Neuregulin-1 gene encodes various isoforms which are generated by transcription from six promoters and alternative splicing of primary transcripts [19, 36]. NRG-1 variants vary with four distinct domains: (1) the N-terminal domain, (2) the EGF-like domain, (3) the juxtamembrane domain, and (4) the C-terminal domain [37]. All functional variants contain EGF-like domain essential for interaction with ErbB receptors [37]. The EGF domain differs in the inclusion of exon A or B, which is referred as to α isoforms if it is derived from exon A or β isoforms derived from exon B [36, 37]. β isoforms have higher affinity for the receptor and tend to produce a more robust, long-term signal as compared to α isoforms [36, 37]. NRG-1 is mainly expressed in neurons and Schwann cells of the nervous system in human [38] and mouse [19], in human/mouse endothelial cells [27, 39] and cardiomyocytes [40], and in human cornea epithelial and stroma cells [37]. They play complicated roles in the development of the nervous and the cardiovascular systems. NRG-1 is initially synthesized as a transmembrane protein; however, it does not reach the cell surface and is expressed as soluble or cytosolic proteins [18] or nuclear protein [41] without a transmembrane domain. We assessed total NRG-1 protein expression level in serum by enzyme-linked immunosorbent assay (ELISA) of ECM (*n* = 5/group). Circulating blood NRG-1 level significantly increased in mice 6 days post infection and declined to a level below that of uninfected mice (controls) 8 days post infection when fatal brain damage occurred (Fig. 5a). This decline in NRG-1 suggests inadequate protection of endogenous serum NRG-1 against severe late-stage factors of ECM indicating that low plasma NRG-1 level correlates with clinical severity of ECM. To determine the relationship between tissue NRG-1 expression especially in brain tissue and malaria infection, total RNA, and whole-cell protein lysate were extracted from ECM mice at different time points. Infection of C57BL/6 mice with *Plasmodium berghei* ANKA (PbA) upregulated NRG-1 mRNA in the brain cortex (Fig. 5b) suggesting that NRG-1 expression may be protective against PbA-induced damage in the brain. Consistent with the levels of mRNA detected by real-time reverse transcription polymerase chain reaction (qRT-PCR) assay (Fig. 5b), total NRG-1 protein levels in the cortex tested by Western blot were significantly elevated in PbA-infected C57 BL/6 mice (Fig. 5c, d).

### NRG-1 attenuated ECM mortality-associated ErbB4/STAT3/AKT signaling

**Fig. 6:**
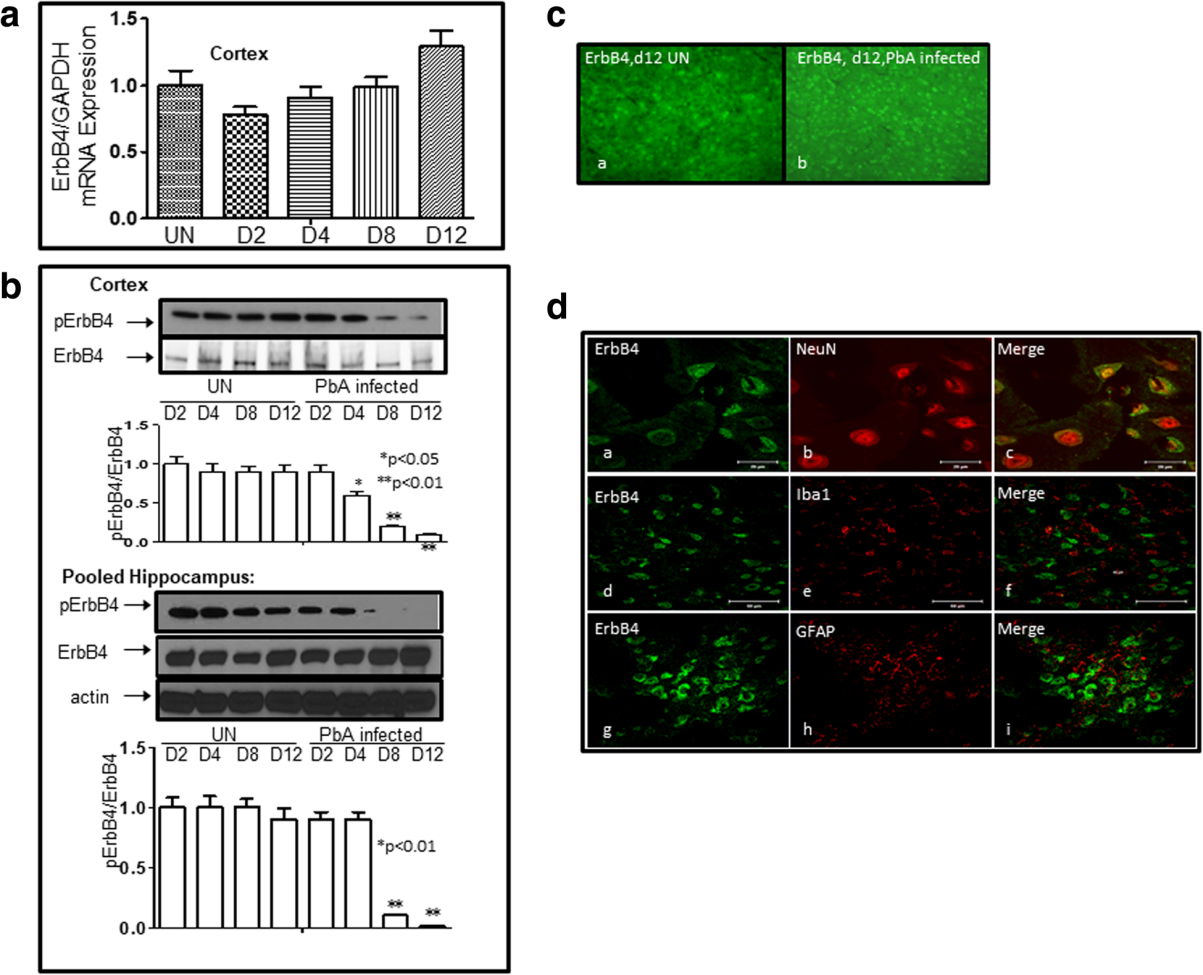
*Phosphorylated ErbB4 (pErbB4) levels decrease in ECM mice.* **a** Total mRNA level of ErbB4 in brain cortex was unchanged during infection with PbA. **b** Protein extracts from the cortex and hippocampus of mice were probed with antibodies against phosphorylated ErbB4 (Tyr1284) and total ErbB4. The data represent one of three independent experiments with similar results. Beneath the Western blot is a densitometric analysis of bands assessed with the ImageQuant program. The data are expressed as fold stimulation relative to appropriate controls and are the mean ± SE of duplicated samples performed in three independent experiments. Total protein level of ErbB4 in the brain cortex was unchanged during ECM, whereas phosphorylated ErbB4 (pErbB4) gradually decreased from day 2 and was hardly detectable at day 12. Expression pattern of pErbB4 in hippocampus was similar to that observed in cortex (*p < 0.05, **p < 0.01, compared to uninfected (UN) sample at D2). **c** Immunofluorescence (IFC) results confirmed the Western blot data, in which total ErbB4 protein was unchanged even 12 days after infection (panels *a*, *b*). **d** IFC data showed that ErbB4 co-localized with neuronal marker-NeuN (panels a–c), but not with microglial cell marker-Iba1 (panels *d*–*f*), or astrocyte marker-GFAP (panels *g*–*i*)

**Fig. 7:**
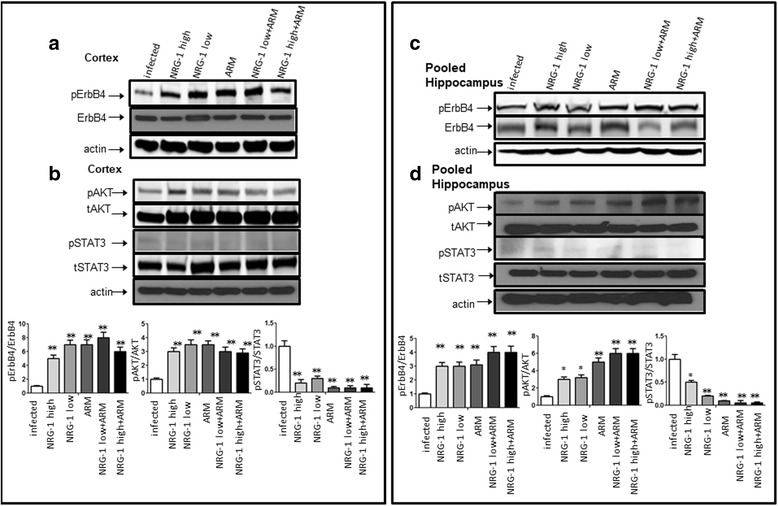
*NRG-1 attenuates mortality associated with ECM through ErbB4/STAT3/AKT signaling.* Protein lysates from different brain regions including the cortex and hippocampus of ECM mice subjected to exogenously infused NRG-1 were probed with antibodies against phosphorylated STAT3, total STAT3, phosphorylated ErbB4 (Tyr1284), total ErbB4 (tErbB4), phosphorylated AKT, and total AKT as indicated. **a** pErbB4 levels increased in PbA-infected mice treated with NRG-1 and ARM individually in brain cortex. **b** Exogenous treatment of NRG-1 and/or ARM inhibited activation of STAT3 and increased activation of pAKT in the brain cortex. Beneath the Western blot is the densitometric analysis of the bands performed with the ImageQuant program. The data are expressed as the fold stimulation relative to appropriate controls and are the mean ± SE of duplicated samples performed in three independent experiments. (*p < 0.05, **p < 0.01, compared to infected sample at first column). **c** pErbB4 levels increased in PbA-infected mice treated with NRG-1 and ARM individually, while there was a robust increase when treated with both NRG-1 and ARM (NRG-1/ARM) in pooled samples prepared from hippocampal tissues. **d** Exogenous treatment of NRG-1 and/or ARM inhibited activation of STAT3 and increased activation of pAKT in brain hippocampus. Beneath the Western blot is the densitometric analysis of the bands performed with the ImageQuant program. The data are expressed as the fold stimulation relative to appropriate controls and are the mean ± SE of duplicated samples performed in three independent experiments. (*p < 0.05, **p < 0.01, compared to infected sample at first column). All the data represent one of three independent experiments with similar results

**Fig. 8:**
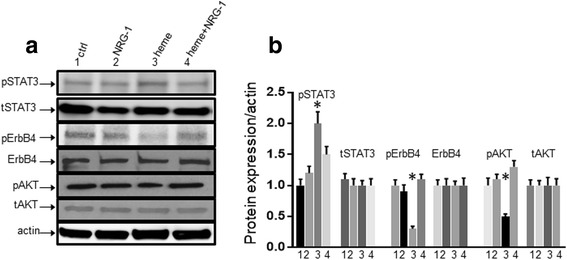
*NRG-1 inhibits STAT3 activation and ErbB4/AKT inactivation* in vitro*.* hCMEC/D3 cells were treated with NRG-1 for 30 min followed by stimulation with 30 μM heme for 24 h. Lysates were extracted and stained with antibodies against STAT3, ErbB4, and AKT as indicated by Western blot. **a** NRG-1 inhibited heme-induced STAT3 activation, increased heme-reduced ErbB4/AKT activation. **b** A densitometric analysis of bands on blots was performed with the Image Quant program (Bio-Rad). The data are expressed as the fold stimulation relative to appropriate controls and are the mean ± SE of duplicated samples performed in three independent experiments. (*p < 0.05)

Next, we investigated whether exogenously infused recombinant human NRG1-beta1 (rhNRG-1) containing EGF-like domain into ECM mice activates the NRG-1/ErbB4 signaling pathway, which is functionally active in ischemia/reperfusion-induced injuries [17]. Real-time quantitative PCR results showed that PbA infection did not change ErbB4 expression in the cortex (Fig. 6a). Whole extracts of mouse cortex tissues were analyzed by Western blots with antibody to the phosphotyrosine residue Tyr1284 within ErbB4 and total ErbB4 antibody. Extracts of cortex showed gradually decreased ErbB4 phosphorylation at Tyr 1284 after 2 days of PbA infection and was hardly detectable by day 12 (upper panel of Fig. 6b). Protein extracts from pooled hippocampus of mice were also probed with antibodies to phosphorylated ErbB4 (Tyr1284) and total ErbB4. The pattern of pErbB4 protein expression from pooled hippocampus tissues was similar to those in the cortex (lower panel of Fig. 6b). Phosphorylated ErbB4 decreased when mice were infected with *P. berghei* ANKA. Consistent with total mRNA expression, levels of total ErbB4 protein did not change in either the cortex or hippocampus. Immunofluorescence (IFC) results confirmed the findings of Western blot analysis whereby total ErbB4 protein remained unchanged 12 days after infection (Fig. 6c, panels a, b). In addition, IFC data showed that ErbB4 co-localized with the neuronal marker-NeuN (Fig. 6d, panels *a*–*c*), but not with microglial cell marker Iba1 (Fig. 6d, panels *d*–*f*), or astrocyte marker glial fibrillary acidic protein (GFAP) (Fig. 6d, panels *g*–*i*). Remarkably, exogenous NRG-1 (at both low (5 μg/kg) and high (25 μg/kg) doses) in combination with/without artemether (ARM) treatment partially restored expression of pErbB4 in both cortex (Fig. 7a) and pooled hippocampus tissues (Fig. 7c). pErbB4 levels increased in PbA-infected mice treated with NRG-1 and ARM individually, while there was robust increase in animals treated with both NRG-1 and ARM (NRG-1+ARM or NRG-1/ARM represents combination of NRG-1 and ARM), particularly in pooled samples prepared from hippocampal tissues (Fig. 7c). These data suggest that the ErbB4 receptor is activated through phosphorylation after the treatment with NRG-1 infusion. We further examined the putative downstream pathways activated by NRG-1/ErbB4. Our interest in signal transducer and activation of transcription 3 (STAT3) and AKT signaling pathways derives from the reported importance of STAT3 in the pathogenesis of cerebral malaria [7, 42] while AKT activation is a typical downstream signal molecule for NRG-1/ErbB4 [17]. Activated STAT3 induced by *Plasmodium* infection was inhibited by treatment with NRG-1 and/or ARM as expected (Fig. 7b, d). In addition, pAKT is upregulated both in brain cortex (Fig. 7b) and hippocampus (Fig. 7d) of PbA-infected mice when treated with NRG-1 and/or ARM compared to untreated control. These results strongly indicate that NRG-1 plays an important role in attenuation of mortality associated with ECM through ErbB4/STAT3/AKT signaling. We also examined whether this mechanism applied to in vitro cell culture systems. To this end, hCMEC/D3 cells were grown and treated with 30 μM of heme with or without 100 ng/ml of NRG-1. Whole cell lysates were isolated under each condition as indicated in Fig. 8, transferred to membranes and probed with pSTAT3/tSTAT3 and pAKT/tAKT antibodies. Consistent with the observation made in our in vivo study the results show that NRG-1 reduced heme-induced STAT3 activation and increases heme-reduced ErbB4 and AKT activation (Fig. 8).

## Discussion

NRG-1 is a member of a family of growth factors which are very important in the development of the heart, mammary glands, and the nervous system [10]. In agreement with the known capacity of NRG-1 to attenuate neuronal apoptosis [17], we demonstrate that NRG-1 protects heme-induced endothelial and neuroglial cell death/apoptosis (Fig. 1). NRG-1 has been shown to be neuroprotective against CNS injury. For instance, in an in vivo experimental trauma model, NRG-1 improved endothelial biological features and reduced BBB permeability and then consequently strengthened BBB structure [43]. In our in vitro BBB model, NRG-1 protected against heme-induced disruption of BBB integrity by strengthening tight junction proteins (Figs. 2 and 3). NRG-1, a growth factor distributes and accumulates in many regions of the brain, including frontal cortex, striatum, and ventral midbrain containing the substantia nigra after systemic application [44]. It targets the entire neurovascular units/cell types such as neuronal cells, oligodendrocytes, endothelial cells, and macrophages to accomplish its potent neuroprotective roles. NRG-1 protects against neuronal death/apoptosis induced by oxygen-glucose deprivation (OGD) [45–47], ischemic reperfusion [48], and deep hypothermic circulatory arrest [17]. It prevents apoptosis of oligodendrocyte progenitor cells caused by OGD [49] and involved in the repair process once the perinatal brain white matter is damaged [38]. It inhibits inflammatory responses either by downregulating pro-inflammatory/inflammatory gene expression in macrophages and resident microglia or hampering macrophage infiltration [45]. Thus, NRG-1 has the potential to be used as a novel therapeutic for treatment of CM since its pathway regulates endothelial cell activities and reduces BBB permeability.

NRG-1 engages the ErbB receptor tyrosine kinases through its EGF-like domain, activation of ErbB receptors impacts cell proliferation, migration, differentiation, and apoptosis in a variety of cell types. The NRG-1/ErbB axis initiates and promotes Schwann cell development and myelination through mobilization of Ca^2+^, activation of nuclear factor of activated T cells (NFAT), activation of kinases such as Ca^2+^-dependent protein kinase C (PKC), ras/extracellular signal regulated kinase ½ (Erk1/2), and phophatidylinositol-3-kinase (PI3K/AKT) [50, 51]. Among these pathways, ErbB4-mediated PI3K/AKT signaling pathway is especially crucial against apoptosis [49]. However, the mechanism by which NRG-1/ErbB interaction protects against cerebral malaria pathogenesis is unknown. For instance, it is unclear how NRG-1 activates its ErbB receptor to protect against cerebral malaria pathogenesis. The extent to which this protection occurs in any specific tissues and regions of the brain remains largely unknown. In the present study, we determined that the ErbB4 protein is inactivated through dephosphorylation in the cortex and hippocampus of mice with advanced stages of ECM (Fig. 6) indicating a diffuse distribution of brain lesions. In contrast, exogenous NRG-1 infusion increases phosphorylated ErbB4 levels in the cortex and hippocampus of infected mice, which subsequently reduces STAT3 activation—a typical pathogenic pathway in CM [7, 42] and increases AKT activation (Fig. 7). Our findings are consistent with Lok’s report which showed that NRG-1 increases pAKT to enhance survival of endothelial cells in in vitro cell cultures [27]. Gene microarray data analysis revealed that NRG-1 downregulated hemeoxygenase-1 (HO-1), a heme scavenger [45], suggesting that NRG-1 may interrupt heme-induced pathogenic signaling pathways in brain endothelial cells. Our findings support the observation that NRG-1 reduces heme-induced STAT3 activation while increasing ErbB4/AKT activation when human endothelial cells are co-treated with heme and NRG-1 in cell culture (Fig. 8).

Although NRG-1 is initially synthesized as transmembrane protein, NRG-1 is cleaved by proteases and the soluble form of NRG-1 impacts significantly the function of the nervous system [18]. Plasma soluble NRG-1 has been detected as a diagnostic biomarker for Alzheimer’s disease [52]. The cause of increased expression of NRG-1 including blood NRG-1 levels after PbA infection is not clear. We speculate that STAT3 could be one of regulators since the promoter of NRG-1 contains a number of STAT3 binding sites. Our in vitro data strongly indicate that increased NRG-1 level reduced damage to the blood brain barrier. So far, no evidence indicates any functional differences between increased NRG-1 during infection and infused NRG-1. However, the bioavailability of infused NRG-1 is very low in circulation; Ford’s group (co-author) reported that the half-life of NRG-1 was short, the plasma t _½_ was about 8 min after exogenous intraarterial injection of NRG-1, while it was present in brains of animals at 20 min post administration and remained at a constant level for up to 4 h post injection [53].

Our data demonstrates that circulating NRG-1 levels correlate with clinical severity of ECM. Interestingly, a previous report by Hama et al. in 2015 [21] indicated that NRG-1 level in plasma did correlate with clinical severity of Parkinson’s disease. Surprisingly, infusion of higher doses of NRG-1 in our study did not produce exponential effects as expected compared to the optimum dose of NRG-1 utilized. The reason for this is unclear. However, Xu et al. [49] found that NRG-1 exerts a dual function in the myelination of oligodendrocytes. It appears that excessive stimulation of NRG-1β signaling may not be beneficial but detrimental for neural function. Low doses of NRG-1 stimulates myelination in vitro [54] while overexpression of NRG-1 in myelinating Schwann cells caused hind limb paralysis, which indicates that over-activation of NRG-1 signaling may generate aberrant Schwann cell activity [49]. We are aware that the neuronal cell scenario is different from that of endothelial cells. However, both cell types are indirectly relevant to HCM and ECM and may explain why higher doses of NRG-1 may fail to produce exponential effects as observed for low doses. Taken together, our results indicate that judicious augmentation of NRG-1 during anti-malaria therapy against CM may be an effective therapeutic approach to increase effectiveness of current anti-malaria therapy against CM.

## Conclusion

Neuregulin-1 (NRG-1) is encoded by the gene *NRG1*, a member of a family of polypeptide growth factors, which is essential for the normal development of the nervous system and the heart. Following our first report of its protective role in experimental cerebral malaria (ECM), we further examined how NRG-1 mechanistically attenuates ECM pathogenesis and reduces mortality. Our results indicate that NRG-1 protects against cell death/apoptosis of human brain microvascular endothelial and neuroglial cells and prevents BBB disruption. Our results also demonstrate that NRG-1 attenuated ECM mortality is associated the activation of ErbB4/AKT and inactivation of STAT3 signaling pathways.

## Methods

### Animal study

Infection of mice with *P. berghei* ANKA was performed as described previously [26]. Mice were selected and randomized into treatment groups after diagnosis with ECM [26] on days 5 to 6 post infection. To determine the therapeutic benefit of NRG-1 on ECM-associated brain damage and mortality and to compare NRG-1 with artemether (ARM) treatment, PbA-infected mice were treated daily via i.p. injection with 50 μl doses of NRG-1 (5 or 25 μg/kg/day, EGF-like domain, R & D Systems, Minneapolis, MN, USA) [NP_039250] or artemether prepared in coconut oil (25 mg/kg/day, Sigma-Aldrich, St Louis, MO, USA) from days 6 to day 11 post infection. The mice treated daily with 50 μl saline solution (i.p.) from days 6 to 11 post infection were used as the controls. Mice were checked several times daily for mortality and signs of ECM neurological symptoms such as ataxia, loss of reflex, and hemiplegia. All murine ECM experiments were terminated 19 days after PbA infection with animals being euthanized accordingly [26].

### Antibodies and reagents

Rabbit antibody against STAT3, phospho-STAT3, rabbit antibody against AKT, phospho-AKT, anti-NeuN, anti-Iba1, and anti-glial fibrillary acidic protein (GFAP) were purchased from Cell Signaling Technology Inc. (Danvers, MA, USA). Polyclonal antibody to β-actin, lipopolysaccharide (LPS), and sodium fluorescein were purchased from Sigma-Aldrich (St. Louis, MO, USA). Rabbit polyclonal antibodies against claudin5, ZO-1, and phosphorylated ErbB4 (monoclonal) antibody were purchased from Abcam (Cambridge, MA, USA). Rabbit polyclonal antibody against occludin and attachment factor (AF) was purchased from Invitrogen (Waltham, MA, USA). Rabbit antibody against VWF was purchased from Dako (Santa Clara, CA, USA). Anti-ErbB4 (polyclonal) and NRG-1 (polyclonal) antibodies were purchased from Santa Cruz (Dallas, TX, USA). All secondary antibodies used for Western blot were purchased from Calbiochem (La Jolla, CA, USA). Hemin (heme) was purchased from Frontier Scientific **(**Logan***,*** UT, USA). Recombinant human NRG1β1/HRG1β EGF domain protein (γhNRG-1) and recombinant human CXCL10/IP-10 protein were purchased from R&D systems (Minneapolis, MN). Recombinant human interleukin-1β (IL-1β) was purchased from Millipore (Billerica, MA, USA).

### Cell culture

The hCMEC/D3 a cell line (Cellutions Biosystems Inc., Ontario, Canada) was derived from human temporal lobe microvessels and were immortalized with hTERT and SV40 large T antigen. The cells have been extensively characterized for brain endothelial phenotype and are a model of human blood brain barrier (BBB) function. hCMEC/D3 cells were cultured at 37 °C with 5% CO2 in endothelial basal medium-2 (Lonza) supplemented with 5% fetal bovine serum (FBS; American Type Culture Collection (ATCC), Manassas, VA, USA), growth factors and other supplements including human recombinant epidermal growth factor (hEGF), hydrocortisone, GA-100 (Gentamicin, Amphotericin-B), human recombinant vascular endothelial growth factor (VEGF), recombinant human fibroblast growth factor-b (hFGF-b), recombinant long R insulin-like growth factor (R3-IGF-1), ascorbic acid, heparin, 100 U/ml of streptomycin, and 100 U/ml of penicillin (Lonza). The cells were harvested and passaged at about 70–90% confluence as described previously [6]. The hCMEC/D3 cells (2 × 10^5^ cells/ml) were seeded in 35-mm tissue culture dish and incubated at 37 °C in 5% CO_2_ for 24–48 h for future use. M059K cells purchased from ATCC were cultured at 37 °C with 5% CO2 in DMEM: K-12 medium.

### Measurement of cell viability by MTT assay

The hCMEC/D3 or M059K cells were seeded at 1 × 10^4^ cells in 100 μl of medium per well into 96-well plates and serum-starved for 24 h, incubating with 100 ng/ml γhNRG-1 for 30 min, followed by exposing to heme at 30 μM or 0.02 μg/ml CXCL10 for 24 h. MTT assay was performed in accordance to the manufacturer’s instructions. Ten microliters of MTT reagent (the ratio of MTT reagent to medium is 1:10) was added into each well and incubated in the dark at room temperature for 2 to 4 h. Absorbance at 570 nm was measured using 650 nm as reference filter by a CytoFluorTM 2300 plate reader and the software CytoFluorTM 2300 v. 3A1 (Millipore Co, Bedford, MA, USA).

### Immunofluorescence staining

Cells grown in monolayer cultures were fixed with 4% paraformaldehyde in phosphate-buffered saline (PBS), permeabilized with 0.2% Triton X-100 and blocked with 10% goat serum prior to antibody staining. Specific primary antibodies were added at 1:100 dilution and incubated at 4 °C overnight. Fluorescent staining was developed using the FITC or Cy3 fluorescence system (Sigma-Aldrich, St. Louis, MO, USA). *TUNEL assay*, in situ cell death detection kit (TMR red; Boehringer-Mannheim, Mannheim, Germany) was used. The sections were incubated with the TUNEL reaction solution for 60 min at 37 °C in the dark. Cover slips were mounted onto slides with Vectashield mounting medium with DAPI (H-1200; Vector Laboratories Inc.). Fluorescent images were collected by using a Zeiss laser scanning microscope (LSM) 510 confocal microscope, and images were captured with LSM software, version 2.3 (Carl Zeiss, Wetzlar, Germany). Apoptotic cells and total cells were counted in ten randomly chosen microscopic fields, and the percentage of apoptotic cells was calculated and compared between the experimental and control groups.

### Enzyme-linked immunosorbent assay (ELISA)

Peripheral blood NRG-1 quantification was assayed by ELISA (MyBioSource, San Diego, CA, USA). At indicated days after PbA infection, a subset of mice (*n* = 5/group) was anesthetized, then 500 μL of peripheral blood was acquired through cardiac puncture. Blood was immediately placed in a biopsy tube with EDTA to prevent clotting. Plasma was obtained by centrifuging the whole blood at 1200 *g* for 15 min at room temperature. ELISA was performed on all samples to quantify plasma levels of mouse NRG-1. The samples were read on a microplate reader (TECAN) at 450 nm.

### Endothelial cell permeability assay

Endothelial cell permeability assay was performed as previously described [28, 43, 55]. In brief, the hCMEC/D3 cells were cultured in complete growth media EGM-2 MV (Lonza, Walkersville, MD, USA). The hCMEC/D3 cells were seeded into the inner surface of attachment factor (AF)/ collagen-coated transwell inserts (24 mm diameter, 0.4-μm pore size polyester filter; Corning, Corning, NY, USA), which were placed in wells of a 6-well plate with human neuroglial/astrocytes (A172 glioma cells). About 7 days after seeding, when hCMEC/D3 cells grown on the inner surface of the insert were confluent (confirmed by testing permeability to media), the cells were serum starved overnight with EBM media without growth supplement, then incubated with IL-1β (10 ng/ml), LPS (1 μg/ml), and heme (30 μM) for 24 h. Thirty minutes prior to addition of IL-1β, LPS and heme, NRG1 (100 ng/ml = 12.5 nM), or vehicle (PBS) was added. After 24 h, media in both upper and lower chambers were removed and replaced with fresh media. Permeability was measured by adding 0.1 mg/ml of Sodium Fluorescein (MW, 376 kDa, Sigma, St. Louis, MO, USA) to the upper chamber. After incubation for 0.5 h, the sample from the lower compartment was collected and 100 μl of sample was measured for fluorescence at excitation 485 nm and emission 535 nm. All three independent experiments were performed in triplicate.

### Real-time RT-PCR analysis

Cell pellets were stored in Trizol reagent and homogenized in fresh Trizol. Total RNA was isolated from cells using an RNeasy Mini Kit (Qiagen, Valencia, CA, USA). Total RNA was quantified using the Nanodrop N-1000 by Agilent Biosystems (Santa Clara, CA, USA). cDNAs were synthesized from the isolated RNA using iScript cDNA Synthesis Kit (Bio-Rad Laboratories, Inc.). Reverse transcription was performed by using random hexamers at 25 °C for 5 min, 42 °C for 30 min, and 85 °C for 5 min. Quantitative PCR was performed using iQ SYBR Green Supermix (Bio-Rad Laboratories, Inc.) in a CFX96 Real-Time PCR System machine (Bio-Rad Laboratories, Inc.). The data was analyzed using CFX96 Real-Time PCR System (Bio-Rad Laboratories, Inc.). Primer sequences for the genes were described in Table 1.

**Table 1:** Primers for real-time polymerase chain reaction

Primer set	Forward primer (5′→3′)	Reverse primer (5′→3′)
NRG-1	tgatcgttgccaaaactacg	accaacagggcgatacagat
ErbB4	tgccataagtcttgcactgg	cgtagggtccatagcacctg
GAPDH	aacgaccccttcattgac	tccacgacatactcagcac

### Western blot

Cells were lysed with lysis buffer (50 mM HEPES, 150 mM NaCl, 1.5 mM MgCl2, 1 mM EGTA, 10% glycerol, 1% Nonidet P-40, 100 mM NaF, 10 mM sodium pyrophosphate, 0.2 mM sodium orthovanadate, 1 mM phenylmethylsulfonyl fluoride, 10 μg/ml aprotinin, and 10 μg/ml leupeptin). Samples were separated by SDS/PAGE, and separated proteins were transferred to nitrocellulose membranes and identified by immunoblotting. Primary antibodies were obtained from commercial sources; these antibodies were diluted at the ratio of 1:1000 according to the manufacturer’s instruction. Blots were developed with Supersignal Pico or Femto substrate (Pierce). A densitometric analysis of the bands was performed with the ImageQuant program (Bio-Rad).

### Statistical analysis

Statistically significant differences were determined using Prism statistical software (Graph Prism 4.03, San Diego, CA, USA). All data were presented as mean ± SEM of at least three independent experiments. For data analysis, unpaired *t* test or one-way ANOVA with Dunnett’s or Bonferroni’s post test was applied. All *P* values resulted from two-sided statistical tests and statistical significance was set at *p < 0.05, **p < 0.01, and ***p < 0.001.

## Abbreviations

ARM: Artemether
BBB: Blood brain barrier
ECM: Experimental cerebral malaria
EGF: Epidermal growth factor
ELISA: Enzyme-linked immunosorbent assay
Erk1/2: Ras/extracellular signal regulated kinase ½
GFAP: Glial fibrillary acidic protein
HCM: Human cerebral malaria
hCMEC/D3: human brain microvascular endothelial cell line
IFC: Immunofluorescence
IRBC: Infected erythrocytes
LPS: Lipopolysaccharide
NFAT: Nuclear factor of activated T cells
NRG-1: Neuregulin-1β
OGD: Oxygen-glucose deprivation
PbA: *P. berghei* ANKA
PI3K: Phophatidylinositol-3-kinase
PKC: Protein kinase C
qRT-PCR: Real-time reverse transcription polymerase chain reaction
SMDF: Sensory and motor neuron-derived factor
STAT3: Signal transducer and activator of transcription 3
TJs: Tight junctions
VWF: Von Willebrand factor VIII
